# Myxoma Virus-Encoded Host Range Protein M029: A Multifunctional Antagonist Targeting Multiple Host Antiviral and Innate Immune Pathways

**DOI:** 10.3390/vaccines8020244

**Published:** 2020-05-23

**Authors:** Masmudur M. Rahman, Grant McFadden

**Affiliations:** Center for Immunotherapy, Vaccines and Virotherapy, Biodesign Institute, Arizona State University, Tempe, AZ 85287, USA

**Keywords:** myxoma virus, MYXV, M029, dsRNA binding proteins, vaccinia E3, oncolytic virus

## Abstract

Myxoma virus (MYXV) is the prototypic member of the *Leporipoxvirus* genus of the *Poxviridae* family of viruses. In nature, MYXV is highly restricted to leporids and causes a lethal disease called myxomatosis only in European rabbits (*Oryctologous cuniculus*). However, MYXV has been shown to also productively infect various types of nonrabbit transformed and cancer cells in vitro and in vivo, whereas their normal somatic cell counterparts undergo abortive infections. This selective tropism of MYXV for cancer cells outside the rabbit host has facilitated its development as an oncolytic virus for the treatment of different types of cancers. Like other poxviruses, MYXV possesses a large dsDNA genome which encodes an array of dozens of immunomodulatory proteins that are important for host and cellular tropism and modulation of host antiviral innate immune responses, some of which are rabbit-specific and others can function in nonrabbit cells as well. This review summarizes the functions of one such MYXV host range protein, M029, an ortholog of the larger superfamily of poxvirus encoded E3-like dsRNA binding proteins. M029 has been identified as a multifunctional protein involved in MYXV cellular and host tropism, antiviral responses, and pathogenicity in rabbits.

## 1. Introduction

Myxoma virus (MYXV) is the prototypic member of the *Leporipoxvirus* genus of the *Poxviridae* family of viruses. Other members of Leporipoxviruses are Shope (Rabbit) fibroma virus (SFV), hare fibroma virus, and Squirrel fibroma virus. The natural hosts for these viruses are Lagomorphs and squirrels. The natural evolutionary host of MYXV is the South American leporid *Sylvilagus brasiliensis*, also called tapeti or brush rabbit. In these animals, MYXV causes a benign cutaneous fibroma, which persists for a few weeks before clearance. The virus in these natural habitats of South America is transmitted by mosquitoes or fleas from one host to another without virus replication in the vector. However, infection of the European rabbit (*Oryctolagus cuniculus*) causes a rapid systemic and lethal disease called myxomatosis [1,2,3,4,5]. The disease myxomatosis is characterized by extensive internal and external lesions and severe immunosuppression accompanied by Gram-negative bacterial infections of the respiratory tract [6]. The natural host of SFV is the eastern cottontail rabbit *Sylvilagus floridanus,* where it causes fibrosarcoma-like tumors [7,8]. Unlike MYXV, SFV causes only benign localized fibromas in European rabbits [3]. These apparent differences between the two viruses have been partially attributed to the fact that MYXV, unlike SFV, can inhibit an apoptotic response in rabbit T lymphocytes and thus is able to replicate and spread via infected leukocytes through the lymphatic system [9]. However, other immunomodulatory proteins in MYXV as well likely contribute to the unique lethality in European rabbits. Due to the extreme lethality of MYXV in European rabbits and the ability of the virus to act as a highly transmissible host-restricted pathogen, MYXV was released in Australia and Europe to control the feral European rabbit populations [10]. Since then, the virus and the host have undergone continuous co-evolution in real-time and is one of the most well-documented natural host-microbial pathogen experiments in evolutionary biology [11]. This virus-host interaction also provides new insights into virus adaptation to new hosts and the evolution of virulence. Since the time MYXV was released in Australia in the 1950s, many virus isolates were sequenced in different phases to track the phenotypic evolution of virulence in both Australia and Europe [10,12,13,14,15]. On the other hand, pathogen pressure also caused changes in the antiviral genes in the host European rabbits across two continents [16]. Interestingly, recent reports describe a new natural recombinant version of MYXV from Spain and Portugal that has leaped from rabbits into hares and caused a novel myxomatosis-like disease in this new host [17,18,19]. It will be of interest to deduce the viral genes in this new virus (designated MYXV-Toledo) that is/are responsible for mediating this host species leap into hares.

The genomes of both MYXV and SFV have been sequenced [20,21]. The double-stranded DNA genome (161.8 kb) of MYXV encodes about 171 functional Open Reading Frames (ORFs), many of which are unique to the leporipoxviruses. The central region of the genome harbors about 100 genes that are conserved among poxviruses, encoding proteins involved in mostly structural and housekeeping functions. The rest of the genes located in the terminal-inverted repeats (TIRs) and the near-terminal unique regions are mostly immunomodulatory genes involved in subverting the host immune system. These genes are predicted to be involved in host-specific functions. Many of these gene functions are studied in MYXV [22,23,24]. In SFV, five MYXV gene orthologs are deleted, which may have caused SFV to become less pathogenic in European rabbits [21]. The immunomodulatory proteins encoded by poxviruses have been classified as viroceptors (virus-encoded mimics of immune receptors), virokines (virus-encoded mimics of immune proteins like cytokines), and intracellular signaling modulators [22,25,26]. This review focuses on one of the vital MYXV-encoded intracellular modulators, and considered a major host range factor, called M029.

## 2. Myxoma Virus M029 Is a Member of the Family of Poxviral E3-Like dsRNA Binding Proteins 

**Figure 1:**
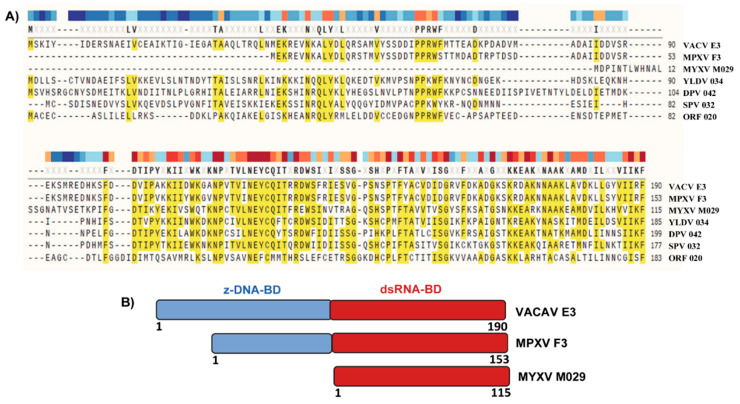
Protein sequence alignment of E3-like proteins. (**A**) Protein sequence alignments of representative E3-like proteins encoded by different poxviruses were generated using the COBALT program (www.ncbi.nlm.nih.gov). (**B**) Schematic diagram of the E3-like protein domains encoded by vaccinia virus (VACV), monkeypox virus (MPXV) and myxoma virus (MYXV).

MYXV-encoded M029 protein is a member of the vaccinia virus (VACV) E3-like poxvirus-encoded dsRNA binding proteins. Among the members of the E3-like proteins, E3 from VACV is the best-studied and characterized protein that antagonizes several dsRNA-activated innate immune signaling pathways [27,28,29,30]. The E3-like proteins encoded by orthopoxviruses are structurally well conserved and composed of a carboxy (C)-terminal dsRNA binding domain (dsRNA-BD) and an amino (N)-terminal Z-DNA binding domain (zDNA-BD), which has also been called the Z-nucleic acid binding domain because of its ability to interact with the Z-forms of both dsDNA and dsRNA [31,32,33] (Figure 1). The only exception in the orthopoxvirus genus is Monkeypox virus (MPXV), where the N-terminal zDNA-BD of E3 protein orthologue (*F3L* gene in MPXV) is truncated by 37 amino acids at the N terminus [34]. However, unlike MPXV, Leporipoxvirus-encoded E3 protein orthologs, like M029 and S029 (the E3 family version encoded by SFV) lack the entire N-terminal Z-DNA binding motif (Figure 1). Members of other genera of poxviruses, for example, yatapoxvirus, swine poxvirus, orf virus, Capripoxvirus, all have intact versions of both the N and C terminal domains. Interestingly, among the poxviruses, Molluscum contagiosum virus (MCV), a human-restricted poxvirus, is unique in that it lacks any ortholog to the E3 protein [35].

At the amino acid level, the dsRNA-BD of the E3-like proteins encoded by distantly related members of the *Poxviridae* family display a higher level of sequence similarity than the N-terminal domain (Figure 1). Thus, it can be predicted that the C-terminal dsRNA-BD might target more conserved and species-independent antiviral pathways. In contrast, the N-terminal domain might target more diverse species-specific antiviral pathways. This domain-specific function was highlighted in multiple studies, where VACV *E3L* was replaced with other unrelated viral or even bacterial genes that encode proteins with a dsRNA-binding motif. For example, VACV lacking *E3L *cannot replicate in HeLa cells; however, expression of the *RNase II* gene product of *E. coli* reversed this phenotype [36]. Another example is influenza virus-encoded NS1 protein, which completely rescued VACV replication in cultured cells in the absence of *E3L* [37]. E3-like proteins encoded by other poxviruses also have been shown to rescue *E3L*-lacking VACV replication in cell culture. For example, the orf virus-encoded E3 ortholog (*OV20.0L* gene), which shares only 31% amino acid identity to VACV E3 protein, can rescue VACV replication in cell culture in the absence of *E3L* [38]. This observation was further extended in a study where the orthologs of E3 from different poxviruses were tested to rescue the function of E3 in VACV [39]. Surprisingly, in this study, not all E3 orthologs rescued the in vitro functions of E3 in VACV. For example, E3 orthologs from swine poxvirus SPV032, and MYXV M029, completely rescued VACV replication in the absence of E3 [39]. On the other hand, the E3 ortholog from YMTV 34L only partially rescued VACV- E3KO; while SPPV34L (sheep and goat poxvirus) did not rescue VACV-E3 KO virus at all. Since M029 can rescue VACV replication in these assays, this suggests that even in the absence of the N-terminal domain, M029 dsRNA-BD might have adapted a broad role in mediating viral host range. In this study, all the tested E3 orthologs had conserved dsRNA binding domains plus N-terminal Z-DNA binding domains (except M029). These results suggest that, although highly conserved at the amino acid level, the diverse poxvirus encoded dsRNA-BD might have adapted host-specific functions to support virus replication in cells of diverse host species.

## 3. Role in Poxvirus Replication

Poxvirus encoded E3-like proteins play an essential role in virus replication in host cell lines. In the case of VACV, the deletion of the *E3L* gene compromised the ability of the virus to replicate in many diverse cell lines [40,41,42,43,44,45]. However, VACV E3 mutant virus was successfully generated and grown in RK13 or BHK21 cell lines derived from rabbit and hamster, respectively [40,46]. Unlike VACV, generation of MYXV and Ectromelia virus (ECTV) E3 orthologs mutant viruses by homologous recombination using flanking sequences and insertion of a reporter gene was not successful in cell lines where these viruses naturally can replicate. In both cases, engineered complementing cell lines were required for constructing and isolating the E3/M029 mutant viruses. In the case of MYXV, the rabbit RK13 cell line engineered to express stably the E3 protein of VACV was used to create the vMyx-M029KO virus [47]. For ECTV, engineered BS-C-1 cells that stably express the E3 protein of VACV were used to generate the ECTVΔE3L virus [48]. Both knockout viruses were only able to be propagated in these complementing cell lines. The purified deletion viruses, when tested for infection and replication in cultured cell lines originated from different species; the viruses were invariably highly defective in replication. For example, vMyx-M029KO virus was not able to replicate at all in tested cell lines originating from human, mouse, or nonhuman primates [47]. The M029-minus mutant virus was also compromised in the viral replication cycle (single step or multistep) in rabbit cell lines like RK13 and RL5 [47]. Similar results were reported in the case of the ECTVΔE3L virus, which was unable to replicate in many mammalian cell lines originating from human, monkey, and mouse sources [48]. This defect in MYXV replication in the absence of M029 and ECTV in the absence of E3 was due to defects that manifest in late gene expression. In the infected cells, the early gene expression and DNA replication allowed the formation of a small viral factory, which indicated the abortive replication of the knockout viruses [48]. The importance of E3 in the life cycle of Modified vaccinia Ankara (MVA), a highly attenuated strain of VACV developed as a live but nonreplicating vaccine, has also been studied [44]. In the absence of *E3L*, MVA was defective in DNA replication, late transcription, and protein synthesis in HeLa cells [44]. These studies thus highlight the importance of E3-like proteins in the viral replication cycle. 

## 4. Role in Pathogenesis

Poxvirus-encoded E3-like proteins are universally essential for the pathogenesis in their native susceptible hosts. Most of the in vivo functions of these proteins are known from the studies on VACV E3. For E3, both the N and C-terminal domains are required for the in vivo pathogenesis of VACV in mice [32,46,49,50]. Similar results were reported with E3 from ECTV. Infection of susceptible BALB/c mice with ECTVΔE3L resulted in the survival of all the mice, whereas infection with wildtype (WT) ECTV and an ECTVΔE3L-revertant virus caused 100% mortality [48]. These results clearly demonstrated that E3 proteins encoded by orthopoxviruses are essential for the virulence in their pathogenic host. Since MYXV causes the lethal disease myxomatosis only in European rabbits, the role of M029 was studied in lab rabbits. In order to understand the role of M029 in MYXV pathogenesis, the vMyx-M029KO virus was intradermally injected in the flank of lab rabbits [47]. Using the same inoculation route, the WT-MYXV- and a M029KO-revertant virus caused 100% mortality of rabbits, whereas the vMyx-M029KO caused only very mild redness and transient swelling at the inoculation site. Unlike the WT-MYXV, no secondary lesion was observed with vMyx-M029KO-infected rabbits [47]. These results suggested that the vMyx-M029KO was highly defective in replication and spread in vivo. Studies with intratracheal injection of VACVΔE3L in C57BL/6 mice exhibited immune cell infiltration and mild edema around the infected bronchi of the lung and very little damage to the lung compared to the WT-VACV infection [46]. Similar results were observed from vMyx-M029KO infection in rabbits, where in the absence of M029, the mutant virus was rapidly cleared by the immune system. In order to test whether the E3-like proteins can complement the in vivo function of VACV E3, several in vivo studies were done in mice [39]. Surprisingly, in all the studies, the E3 protein orthologs were not able to rescue the full pathogenesis of VACV in the host. For example, in the absence of VACV E3, the orthologous proteins SPPV34, YMTV34, SPV032 and M029 were not able to restore the in vivo pathogenesis of VACV in the BALB/c mice when viruses were delivered intranasally [39]. However, as mentioned before, in vitro, both M029 and SPV032 restored the replication of the VACVΔE3L virus. Similar results were reported when the orf E3 protein was expressed in VACV lacking *E3L* [39]. The orf E3 expressing VACV was more than 1000-fold less pathogenic than WT VACV [38]. These results suggest that E3 orthologous proteins encoded by different poxviruses have evolved to be adapted for more host-specific functions for pathogenesis in specific host species, which are yet to be identified.

## 5. Regulation of Host Antiviral Signaling Pathways

### 5.1. Sequestration of dsRNA

**Figure 2:**
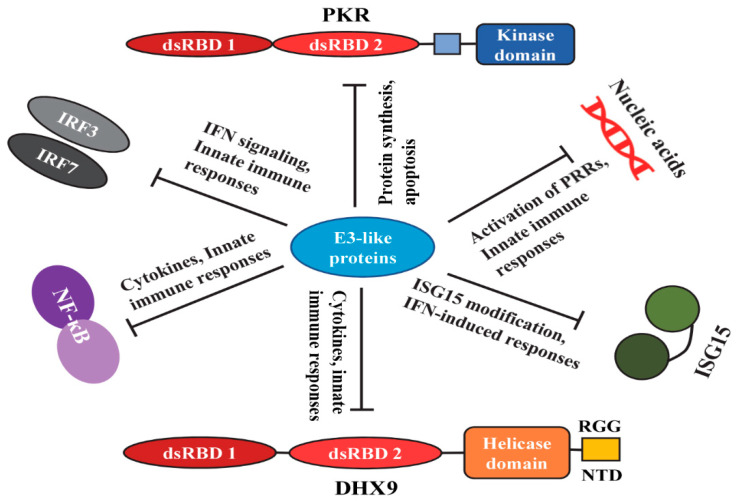
Schematic representation of the known signaling pathways and proteins targeted by poxvirus E3-like proteins. Poxvirus encoded E3-like proteins directly interact with multiple host proteins and modulate diverse signaling pathways.

Many viruses produce dsRNA during their replication cycle in the infected cells. The DNA genome containing poxviruses makes dsRNA because of the synthesis of complementary overlapping transcripts that can anneal to form dsRNA. Indeed, VACV accumulates large amount of cytoplasmic dsRNA during the late replication cycle [51,52,53,54]. These viral dsRNAs function as PAMPs and therefore host antiviral pathways can be activated to alert the innate immune system against the virus infection [55,56,57]. To counteract the host responses to viral dsRNA, viruses either sequester it, degrade it, or interfere with the host cell sensing machinery or effector pathways. Thus, the production of less dsRNA (presumably by minimizing late run-on transcription compared to VACV) can be a strategy for some poxviruses such as MPXV, ECTV, and MYXV to subvert host activation of innate immune responses [58,59]. As an additional measure, these viruses also encode E3-like dsRNA binding proteins (Figure 2). The key pathways that are activated in response to viral dsRNA include type I interferon (IFN), oligoadenylate synthetase (OAS)-RNase L, and protein kinase R (PKR). To overcome the activation of these pathways, viruses have acquired multiple proteins and mechanisms that directly/indirectly target these proteins and disrupt the signaling pathways. For example, VACV encodes multiple proteins such as E3, K3, and K1; all are reported to target dsRNA-induced pathways. However, among these proteins, only E3 orthologs, including M029, have a dsRNA binding motif. E3 was initially characterized as both a dsRNA binding protein and an inhibitor of PKR activation [27,60,61,62]. The C-terminus single dsRNA-binding motif binds dsRNA in a sequence-independent manner [27,32]. The formation of the dimer in solution allows an E3 high-affinity interaction with dsRNA [63]. The C-terminal dsRNA binding domain is required for pathogenesis. However, to understand whether dsRNA binding is essential for the biological functions of the E3 protein, the entire dsRNA-BD amino acids were substituted with alanine. The results suggested that most of the alanine-substituted E3 mutants that were defective in dsRNA binding were also defective in biological functions of E3. However, some E3 mutants were identified that were essential for dsRNA binding but not essential for the biological functions of E3 [64]. The presence of a high degree of similarity and identity among the dsRNA-BD of E3-like proteins from diverse poxviruses might give similar results.

### 5.2. Inhibition of PKR and Determination of Virus Host Range

The IFN-induced dsRNA dependent protein kinase (PKR) is a crucial component of the host innate immunity against virus infection, replication, and spread. PKR is a serine-threonine kinase composed of two N-terminal dsRNA-BD and a C-terminal kinase domain [65]. It was shown that IFN-treated and VACV-infected cells have a translational block of viral and cellular mRNAs [66,67]. This block in protein synthesis was due to the production of dsRNA by VACV and subsequent activation of PKR [68,69,70]. Apart from viral dsRNA, PKR is also activated due to diverse cellular stresses [71]. In the presence of an adequate stimulus, PKR activation and autophosphorylation leads to the phosphorylation of the alpha subunit of eukaryotic translation initiation factor 2 (eIF2α), which eventually activates global protein synthesis shut down and induction of apoptosis [72]. Among many other cellular functions, PKR also has a role in signal transduction and transcriptional control through the IκB/NF-κB pathway [73,74]. Many pathogens, including viruses, encode proteins that directly interact with PKR or different aspects of the PKR signaling pathway to stop the process [75,76,77]. Poxvirus-encoded E3 proteins not only sequester dsRNA, but also inhibit PKR by direct interaction, leading to the formation of heterodimers [63,69] (Figure 2). The fact that PKR is the key target of E3 ortholog proteins is proven by the observations that the replication defect of *E3L*-knockout viruses can be rescued using cell lines with reduced or no PKR expression [78]. This was shown in multiple poxviruses lacking expression of E3 orthologs [47,48,78]. Absence of PKR restored the viral late protein synthesis and progeny virus formation in cell lines where the *E3L* mutant poxviruses were not able to replicate. For example, PKR knock-down or knockout restored MYXV replication in human cell lines in the absence of M029 [47]. Similar results were also reported for the ECTVΔE3L virus [48]. For PKR inhibition, both the N- and C-terminal domains of E3 protein are required [63]. Although previous studies suggested that inhibition of PKR and interaction with dsRNA are closely linked, detailed E3 mutational studies showed that these two functions of the E3 protein could be separated [64]. In the case of MYXV-M029 and MPXV-F3, where a full or partial N-terminal domain of E3 is missing, deletion and mutational studies in these proteins might further shed light on the role of dsRNA domain and adjacent sequences in the inhibition of PKR. Since PKR is the key target of E3 protein orthologs, the ability to inhibit PKR from different species often determines the in vitro cellular tropism of poxviruses [79,80]. Inhibition of PKR is also crucial for the regulation of type I IFN and pro-inflammatory cytokines involved in the activation of innate immune responses [30,81]. For VACV, it was demonstrated in human A549 cells that knock-out of both PKR and RNase L pathways completely rescued the replication of E3 mutant virus, suggesting that both of these pathways are successfully inhibited by VACV [82]. In vivo, both PKR and RNase L single knockout C57BL/6 mice showed a more rapid and increased disease severity, even with a lower dose of WT-VACV and a higher dose of VACVΔE3L virus [46]. These results again confirmed that PKR and RNase L are the crucial target for poxvirus replication and pathogenesis. Apart from E3, poxvirus-encoded protein K3 orthologs (M156 in MYXV), an eIF2α homolog, act as a pseudosubstrate for PKR to inhibit activation of PKR [83]. 

## 6. Myxoma Virus Interference of the Type I IFN Pathway and the Role of M029 

Type I IFNs are produced and released from cells in response to virus infection or other stimulation. They play a major role in activating host antiviral innate and adaptive immune responses. Type I IFNs bind to specific IFN-receptors on cells to trigger signaling pathways that result in the expression of genes called IFN-stimulated genes (ISGs). The expression of ISGs render the cells resistant to subsequent virus infection. Poxviruses have acquired different mechanisms to inhibit production of IFNs, IFN signaling and function of ISGs [25,26,84]. However, among the members of poxviruses, this ability to interfere with type I IFN signaling and responses varies widely. For example, VACV can inhibit almost every aspect of type I IFN signaling in cell lines originating from different species [80,84]. MYXV, on the other hand, is very species-specific in terms of inhibition of type I IFN and may explain why MYXV is restricted to only lagomorphs. This was first reported in a study using primary mouse embryonic fibroblasts (pMEFs) [85]. MYXV infection of pMEFs activated ERK signaling which activated IRF3 and eventually type I IFN induction. This early induction of type I IFN prevented virus gene expression and replication in pMEFs [85]. The role of ERK was further confirmed by inhibition of ERK signaling or disruption of STAT1-mediated IFN signaling, both of which completely rescued MYXV replication in the pMEFs. This was further confirmed in vivo in mice where MYXV did not cause any disease. However, STAT1 deficiency made the mice highly susceptible to lethal MYXV infection [85]. Thus, the ERK-IFN-STAT1 signaling cascade is the key to protect mice against MYXV infection. Although this signaling cascade is highly conserved across the species, MYXV can completely inhibit this cascade and cause lethal disease in rabbits. So, this suggests that MYXV-encoded proteins that target this pathway are highly host- and species-specific.

The IFN pathway is also a vital restriction determinant of MYXV replication in primary human fibroblasts [86]. In contrast to pMEFs, when they were spontaneously immortalized (iMEFs) by multiple passages, these immortalized cells now fully supported productive MYXV infection [87]. In iMEFS, the IFN signaling pathway was intact; however, the MYXV infection-induced type I IFN induction was compromised, possibly at the level of the initial sensing of the virus infection [87]. These findings were further confirmed by the observation that unlike pMEFs, the phosphorylation of ERK1/2 was not detectable, which was further confirmed that there was little or no induction of type I IFN production in the iMEFs [87]. These results indicated that pathogen-associated molecular patterns (PAMPs) sensing of MYXV in pMEFs is likely a key step in induction of type I IFN that is completely lost during the immortalization process. These studies thus confirmed that in immortalized or transformed nonrabbit cells (such as NIH3T3 or iMEFs), the type I IFN-induced antiviral state can restrict MYXV replication. In a later study, this observation was further extended by comparing cells derived from rabbits, humans and mice [88]. Representative cell lines from all these species were able to induce an antiviral state when treated with type I IFN. But surprisingly, when they were infected with MYXV, the viral ability to infect the various type I IFN-treated cells was dramatically different. In rabbit cells, despite the induction of antiviral state, the virus was able to replicate completely even in the presence of the fully-formed antiviral state. In human cells, on the other hand, the virus was able to partially replicate, whereas in the mouse cell line the virus was not able to replicate to any measurable extent [88]. This suggests that MYXV’s ability to counteract the effector functions of the type I IFN induced antiviral state is highly species-specific.

This species-specific ability to inhibit the operational IFN-induced antiviral state was completely dependent on the presence of the M029 protein [88]. In the absence of M029, the vMyxM029KO virus was not able to inhibit the type I antiviral state in rabbit cells. Even in the human cells, the partial inhibition of the antiviral state was also completely lost in the absence of M029 [88]. These results indicated that unlike VACV E3, which is relatively promiscuous in terms of host species specificity, the MYXV M029 retained rabbit and to some extent human-specific inhibitory activity against a type I IFN-induced antiviral state. Furthermore, it was confirmed that this antiviral state was independent of the host cell PKR [88]. In the absence of PKR expression, the vMyxM029KO was inhibited by the antiviral state in rabbit and human cells. This suggests that ISG(s), in addition to PKR, remain to be identified as likely targets of M029 for the inhibition of the type I IFN antiviral state in rabbit and human cells. Based on the studies from VACV, the candidate ISGs could be ISG15, OAS, or IFITs [89,90,91]. Among these ISGs, ISG15 is directly targeted by VACV E3 (Figure 2). In addition, MYXV lacked a homolog of VACV B18, a soluble inhibitor of type I IFNs that directly binds to type I IFN and inhibits type I IFN-induced signaling. B18 showed broad species specificity and can inhibit type I IFNs from humans, rabbits, mice, rats, and bovine animals [92,93,94]. The absence of such an inhibitor also restricts MYXV capacity to infect species other than the leporids.

## 7. Other Cellular Targets of E3 and M029

E3 orthologs are one of the critical proteins in host immune modulation by targeting different cellular pathways. However, many cellular- and host-specific protein targets are yet to be identified. Expression of tagged E3-like proteins in the infected cells and mass-spectrometry followed by co-immunoprecipitation (co-IP) will allow identification of unknown interacting host proteins. To identify the cellular interactions of M029, a recombinant MYXV expressing a V5-tagged M029 protein under the native promoter was constructed [47]. Co-IP using the anti-V5 antibody allowed the pull-down of proteins that interacted with M029 during MYXV infection. Using this approach, cellular DHX9 was identified as an additional cellular interaction of M029 in human cancer cells [47]. However, unlike PKR knockdown, the DHX9 knockdown did not rescue the replication of MYXV in the absence of M029. In another study, DHX9 was identified as a host antiviral RNA helicase in human cancer cell lines where MYXV replicated poorly, for example, pancreatic cancer cell line PANC-1 and renal cancer cell line 786-0 [95]. In these cell lines, knockdown of DHX9 significantly enhanced MYXV replication. In addition, DHX9 has proviral functions based on its requirement for the replication of multiple RNA viruses [96]. DHX9 also functions as a sensor for induction of innate immune responses against PAMPs and viruses in immune cells [97,98,99]. Thus, both RNA and DNA viruses exploit DHX9 to optimize their replication. Like MYXV M029, the VACV E3 protein has been shown to interact with DHX9 in human monocytes, such as THP1 cells. Unlike dendritic cells, in this cell line, the nucleic acid-sensing role of DHX9 was not found, suggesting the cell-type-specific function of DHX9 as a viral PAMP sensor. However, in these cells, DHX9 played a role in IL-6 induction, which was directly antagonized by VACVC E3 [100] (Figure 2). These results suggest that by direct interaction with DHX9, E3 might suppress innate immunity by reducing the synthesis of selected innate defense cytokines.

## 8. Vaccine Vector and Gene Delivery

Several members of the poxvirus family have been developed as a vaccine platform because of their host restriction. For example, VACV strain MVA, fowlpox, and canarypox (members of the genus avipoxvirus), have been developed as vaccine vectors for different host species [101,102,103,104,105]. Since MYXV only infects leporids but still can bind to most mammalian cells of any species, and causes abortive infections in primary nonrabbit cells, it has the potential to be developed as safe vaccine platform for hosts outside of rabbits. As a vaccine vector platform outside of leporids, the safety and efficacy of MYXV has been tested by vaccination of cats against feline calicivirus [106,107] and in sheep against bluetongue virus [108,109]. Also as a rabbit-specific vaccine platform, recombinant attenuated strains of MYXV that do not cause myxomatosis have been used for rabbit vaccination against myxomatosis and rabbit hemorrhagic disease [110]. The M029-deleted MYXV could be a safe alternative for the development of the attenuated vaccine platform in rabbits, and possibly other species as well. VACV E3L mutants are investigated as a vaccine platform due to their greater safety profile in comparison to WT-VACV [111,112]. The New York City Board of Health (NYCBH) strain of VACV was compared to NYCBH deleted for *E3L* (NYCBHΔE3L) in a rabbitpox virus (RPXV) challenge study in the rabbit model [113]. Both the WT-NYCBH and NYCBHΔE3L vaccines completely protected rabbits against a lethal challenge of RPXV. However, unlike a single dose of WT-NYCBH, two doses of NYCBHΔE3L were required to prevent weight loss, fever, and clinical symptoms following challenge with RPV [113]. The attenuated NYCBHΔE3L was also tested in cynomolgus macaques, which were challenged with heterologous MPXV. However, in this model, NYCBHΔE3L provided partial protection against the MPXV challenge [114]. Unlike different VACV strains, MYXV has not been widely tested as a vaccine candidate, other than the examples cited above. However, the inability to cause disease outside the leporids and the capacity to generate robust immune responses in murine or human dendritic cells suggests that MYXV could readily be adapted as a vaccine platform in humans [115,116]. In this context, the M029-deleted MYXV could be a candidate vaccine platform for this purpose. 

## 9. Conclusions and Future Directions

Among the many members of poxviruses with restricted host tropism, MYXV is by far the most well-studied model to understand the regulation of viral pathogen-host species interactions. Since MYXV was released in Australia and Europe in the 1950s to control feral rabbit populations, it also serves as a real-time model to study co-evolution of virus and host in the wild. MYXV encodes many unique proteins which have, or are predicted to have, host-restricted immunomodulatory functions. Studies of MYXV-encoded genes by genetic manipulation have allowed greater understanding of how poxviruses like MYXV can interact with, and subvert, the host immune system in a species-specific manner. Due to this natural host restriction, coupled with the acquired ability to infect transformed or cancerous cells originating from different species outside of the rabbit, MYXV is currently being developed as an oncolytic virus for the treatment of different types of human cancers. To mediate the tropism of MYXV for cancer cells, the virus-encoded immunomodulatory proteins clearly play an important role. However, in an intact nonrabbit host with a functional immune system, the operational activities of these immunomodulatory proteins can have a much different outcome than in rabbits. In this context, for the clinical development of MYXV, it is important to understand the function of these proteins in both lagomorphs and non-lagomorphs. M029 is an essential immune regulatory protein encoded by MYXV that mediates the virus tropism in both primary rabbit cells and in nonrabbit cancer cells. As a member of the E3 family of poxvirus host range proteins, M029 retains vital features important for MYXV tropism in all rabbit cells and many nonrabbit cancer cells by the regulation of the early innate immune responses in the infected cells. To further understand the function(s) of M029 and other E3-like proteins, the identification of additional cellular interactions will be crucial.
